# The autonomic nervous system in septic shock and its role as a future therapeutic target: a narrative review

**DOI:** 10.1186/s13613-021-00869-7

**Published:** 2021-05-17

**Authors:** Marta Carrara, Manuela Ferrario, Bernardo Bollen Pinto, Antoine Herpain

**Affiliations:** 1 Department of Electronics, Information and Bioengineering, Politecnico di Milano, Milan, Italy; 2 Department of Acute Medicine, Geneva University Hospitals, Geneve, Switzerland; 3 Geneva Perioperative Basic, Translational and Clinical Research Group, Geneva University Hospitals, Geneve, Switzerland; 4 Department of Intensive Care, Erasme University Hospital, Université Libre de Bruxelles, Brussels, Belgium; 5 Experimental Laboratory of Intensive Care, Erasme Campus, Université Libre de Bruxelles, Brussels, Belgium

**Keywords:** Sepsis, Septic shock, Autonomic nervous system, Autonomic dysfunction, Dysautonomia, Sympathetic overstimulation, Baroreflex, Tachycardia, Vagal stimulation, Desensitization

## Abstract

The autonomic nervous system (ANS) regulates the cardiovascular system. A growing body of experimental and clinical evidence confirms significant dysfunction of this regulation during sepsis and septic shock. Clinical guidelines do not currently include any evaluation of ANS function during the resuscitation phase of septic shock despite the fact that the severity and persistence of ANS dysfunction are correlated with worse clinical outcomes. In the critical care setting, the clinical use of ANS-related hemodynamic indices is currently limited to preliminary investigations trying to predict and anticipate imminent clinical deterioration. In this review, we discuss the evidence supporting the concept that, in septic shock, restoration of ANS-mediated control of the cardiovascular system or alleviation of the clinical consequences induced by its dysfunction (e.g., excessive tachycardia, etc.), may be an important therapeutic goal, in combination with traditional resuscitation targets. Recent studies, which have used standard and advanced monitoring methods and mathematical models to investigate the ANS-mediated mechanisms of physiological regulation, have shown the feasibility and importance of monitoring ANS hemodynamic indices at the bedside, based on the acquisition of simple signals, such as heart rate and arterial blood pressure fluctuations. During the early phase of septic shock, experimental and/or clinical studies have shown the efficacy of negative-chronotropic agents (i.e., beta-blockers or ivabradine) in controlling persistent tachycardia despite adequate resuscitation. Central α-2 agonists have been shown to prevent peripheral adrenergic receptor desensitization by reducing catecholamine exposure. Whether these new therapeutic approaches can safely improve clinical outcomes remains to be confirmed in larger clinical trials. New technological solutions are now available to non-invasively modulate ANS outflow, such as transcutaneous vagal stimulation, with initial pre-clinical studies showing promising results and paving the way for ANS modulation to be considered as a new potential therapeutic target in patients with septic shock.

## Introduction

Sepsis is defined as life-threatening organ dysfunction caused by a dysregulated host response to infection [1]. Although guidelines for septic shock resuscitation have been extensively revisited over recent years [1–4], they have not included the potential role of autonomic nervous system (ANS) dysregulation.

Several studies have documented altered sympathetic activity with the progression of circulatory shock severity, using direct measures of sympathetic activity during surgical or pharmacological interventions in animal models [5, 6], or indirect estimations of autonomic activity, such as heart rate variability (HRV), blood pressure variability (BPV), or baroreflex sensitivity, in patients/animal models [7–12]. ANS response can occur rapidly, over seconds to minutes, to restore homeostasis. Clinical signs of ANS dysregulation may therefore be an early warning sign of an imminent decline in cardiovascular function, occurring before any deterioration in more global markers, such as a decrease in arterial blood pressure, which may occur too late to enable preventive action to be taken. Including variables associated with ANS regulation of the cardiovascular system, along with variables traditionally displayed on bedside monitors, has already been proposed as a useful tool for anticipating potential clinical deterioration in critical care settings [13–16]. One example of this approach is the HeRO (Heart Rate Observation) monitor, which displays a score indicating the risk of an infant deteriorating with sepsis in the next 24 h to prompt earlier interventions [17]. Display of this HeRO score resulted in a 22% relative reduction in mortality in a randomized clinical trial in neonatal intensive care unit (ICU) patients [17]. Similarly, the hypotension prediction index (HPI), based on dynamic changes in the variability and complexity of variables estimated from the arterial pressure waveform, has recently been proposed [18], and clinical trials are ongoing to validate the index and evaluate its impact on outcomes.

During the resuscitation phase in septic shock, little attention has been given so far to the assessment of ANS-mediated control of the cardiovascular system. However, the ANS has a key role in maintaining cardiovascular homeostasis in both physiological and pathological conditions, and its dysfunction during sepsis and septic shock has been extensively demonstrated (Table 1).

**Table 1:** Relevant clinical and pre-clinical investigations supporting autonomic dysfunction in response to sepsis and septic shock (or endotoxic shock)

Markers	Main results	Type of studies	References
Catecholamines/ neuroendocrine mediators	Endogenous catecholamine increase after a sympathetic insult (e.g., head-up tilt) was larger in sepsis survivors than in non-survivors. Mortality and morbidity were higher in patients with higher and more prolonged catecholamine burden	Clinical studies	Benedict and Rose [44], Ostrowski et al. [28], Boldt et al. [27], Schmittinger et al. [29]
	In the late phase of sepsis, animals showed reduced vasopressin release. Animal models of sinoartic denervation and bilateral carotid neurotomy showed an increase in catecholamines and TNF-alpha release	Animal studies	Pancoto et al. [8], Shi et al. [9], Nardocci et al. [57]
Adrenergic receptors	Endotoxemia caused systemic α1-receptor downregulation in all organs investigated; tissue concentrations of interleukin-1β and TNF-α were markedly increased	Animal study	Bucher et al. [47]
	Patients presenting with sepsis or septic shock had extended deficits in β-adrenergic post-receptor signal transduction	Clinical study	Bernardin et al. [48]
HRV, BPV, BRS and chemoreflex sensitivity	BRS, chemoreflex sensitivity, and almost all HRV indexes were attenuated in MOF patients. Patients in whom organ function (SOFA score) increased significantly showed an increase in mean BP value and LF power in BP time series	Clinical studies	Schmidt et al. [7], Pontet et al. [77], Carrara et al. [84]
	Reduced HRV indexes preceded sepsis-induced macro-hemodynamic alterations. HRV reduction was associated with pronounced parasympathetic inhibition and a sympatho-vagal balance shift. BRS was reduced after fecal peritonitis; overall variability, LF power, LF/HF ratio of HR, and LF power of MAP were all reduced. Denervated rats had the lowest BRS and survived the shortest time after the peritonitis. Endotoxic shock induced a very rapid impairment in baroreflex function, independent of the BP level. Septic shock induced an inversion of the physiological pulse pressure amplification; aortic time constant tau, aortic compliance and TPR were decreased; BRS and aortic time constant tau alterations were correlated	Animal studies	Pancoto et al. [8], Jarkovska et al. [78], Shi et al. [9], Shen et al. [56], Nardocci et al. [57] Radaelli et al. [5], Carrara et al. [82]
Autonomic centers	Septic shock is associated with neuronal and glial apoptosis within the autonomic centers, which is strongly associated with endothelial iNOS expression	Post mortem patient study	Sharshar et al. [55]

*BRS* baroreflex sensitivity, *TPR* total peripheral resistance, *HRV* heart rate variability, *MAP* mean arterial pressure, *LF* low frequency, *HF* high frequency

The objective of this narrative review is therefore to discuss the recent evidence, from studies using standard or advanced monitoring methods and mathematical models, to support the importance of ANS monitoring in patients with sepsis and septic shock and the potential for the ANS to become an important therapeutic target in this context.

## The autonomic nervous system

### The parasympathetic and sympathetic branches

**Fig. 1:**
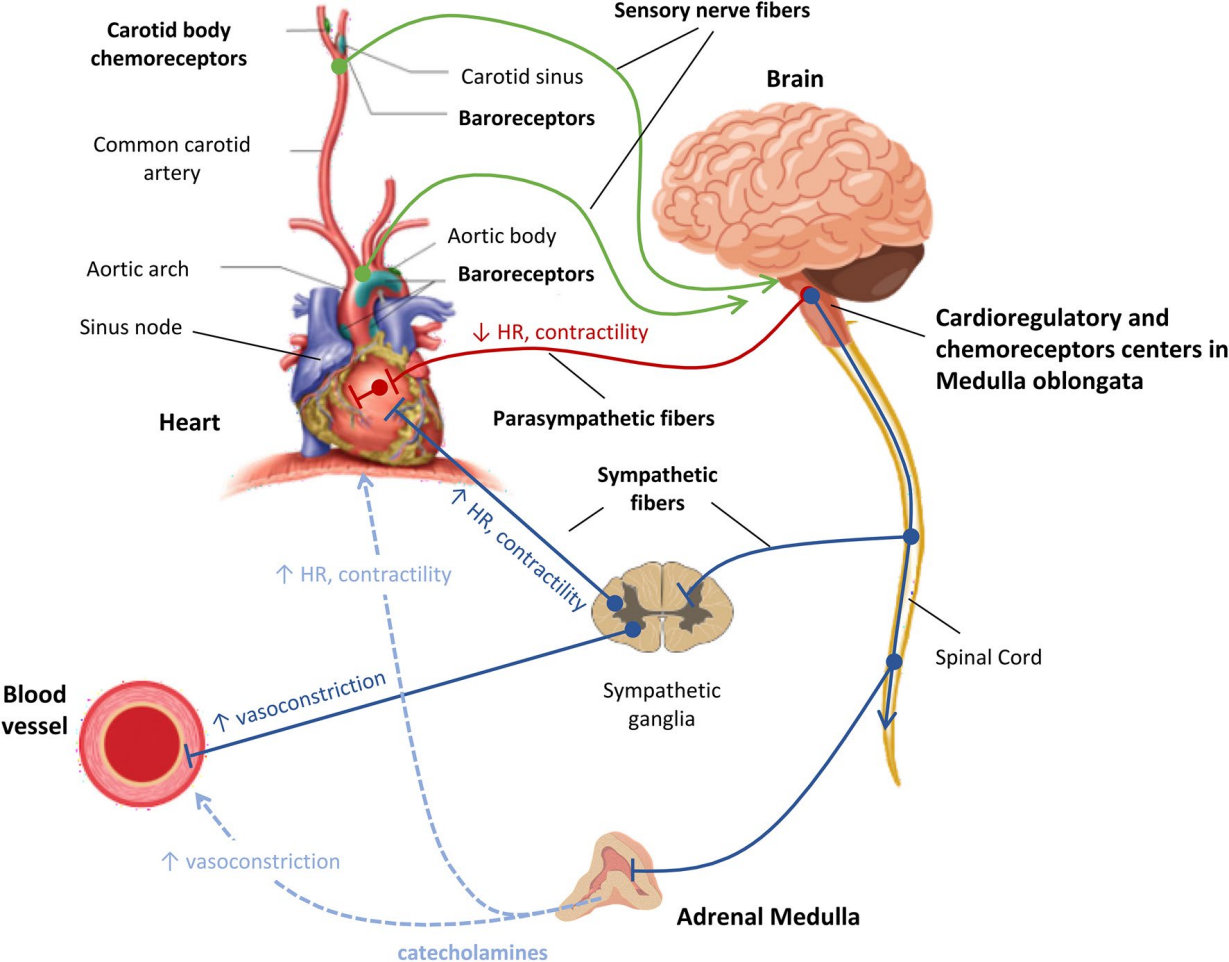
Illustration of the short-term ANS regulatory mechanisms of the cardiovascular system. The arterial baroreceptors, usually known as high-pressure baroreceptors, are mainly located in the aortic arch and carotid sinuses. Cardiopulmonary baroreceptors—also known as volume-receptors or low-pressure baroreceptors—are located in the atria, ventricles, vena cava, and pulmonary vessels. The chemoreceptors are located both peripherally (carotid bodies and aortic arch) and centrally. The vasomotor center hosts the circulatory regulation and is part of the medulla oblongata located in the brainstem, next to the nucleus of the solitary tract that receives sensory nerves signals through the glossopharyngeal and the vagus nerves (green lines). The SNS fibers (blue lines) originate from the medulla oblongata and emerge from the spinal cord's upper thoracic segments as pre-ganglionic neurons, ending inside the sympathetic chain ganglia located next to the vertebral column. SNS post-ganglionic neurons leave the sympathetic chain ganglia towards their target organs, namely the heart, the vessels, and the adrenal glands. Note that adrenal medulla sympathetic activation also induces epinephrine and norepinephrine release (dashed light blue lines) into the bloodstream. The PNS fibers (red lines) also originate from the brain stem and are incorporated as pre-ganglionic neurons in the vagal nerve, ending on the parasympathetic cardiac ganglia lying in the heart fat pad. Very short post-ganglionic neurons arise from these parasympathetic cardiac ganglia towards the right and left atrium, the atrioventricular node, the interatrial septum, the ascending aorta, and the pulmonary trunk. During septic shock, hypotension and the inflammatory reaction inhibit the vagal centers—inducing vagal outflow reduction—whereas the sympathetic pathway is stimulated. Sepsis, therefore, provokes a striking imbalance in ANS activity with a shift towards the SNS

The ANS is part of the central nervous system (CNS) and provides unconscious control of vital physiological functions, ensuring body homeostasis. The ANS is centrally regulated by the hypothalamus, which acts as an integrator for autonomic functions. The two efferent branches of the ANS system are the parasympathetic (PNS) and sympathetic (SNS) nervous systems, both of which are characterized by two types of neuron: pre-ganglionic and post-ganglionic fibers (Fig. 1).

The PNS is composed of several nerves, e.g., the glossopharyngeal, the vagus, and the pelvic splanchnic nerves. Unlike the SNS, all the pre- and post-ganglionic nerves of the PNS are exclusively cholinergic, activating nicotinic receptors in the synapses between pre- and post-ganglionic neurons (as for the SNS), but muscarinic receptors at the target organ level. The PNS innervates the heart through the vagus nerve until it reaches the parasympathetic cardiac ganglia lying in the fat pad on the heart’s surface, from which arise very short post-ganglionic neurons towards the heart itself. Hence stimulation of the PNS and vagus nerves directly influences only the activity of the heart, with negative chronotropic and inotropic effects, but can also modify the blood pressure (BP) as a secondary effect of cardiac output (CO) regulation [19].

The pre-ganglionic SNS neurons originate from the thoracolumbar region of the spinal cord. They are all cholinergic and travel to the paravertebral sympathetic ganglia, activating nicotinic receptors in the synapses between pre- and post-ganglionic neurons. These latter are usually longer neurons, extending through the body until they reach the surface of their target organs. They are typically adrenergic, releasing norepinephrine on the target organs’ adrenergic receptors. Moreover, SNS can also promote epinephrine secretion in the bloodstream by the adrenal medulla stimulation, acting as a circulating hormone.

### Lifecycle of adrenergic receptors

Adrenergic receptors are characteristic only of the SNS, and can be divided into two types, α-receptors and β-receptors, which can be further divided into subtypes and subclasses [19, 20]. From the cardiovascular standpoint, α1-receptors are mostly located on the blood vessels and when activated are responsible for vasoconstriction; β1 (and β2) receptors are mostly present on the heart (the β1/β2 ratio in the myocardium is around 3:1 to 4:1) where their stimulation mediates positive chronotropic, dromotropic and inotropic effects. At the vessel level, β2-receptor stimulation causes vasodilation [20]. Of note, in the CNS, presynaptic α2-receptor activation inhibits the release of norepinephrine and thus produces a global sympatholytic action.

**Fig. 2:**
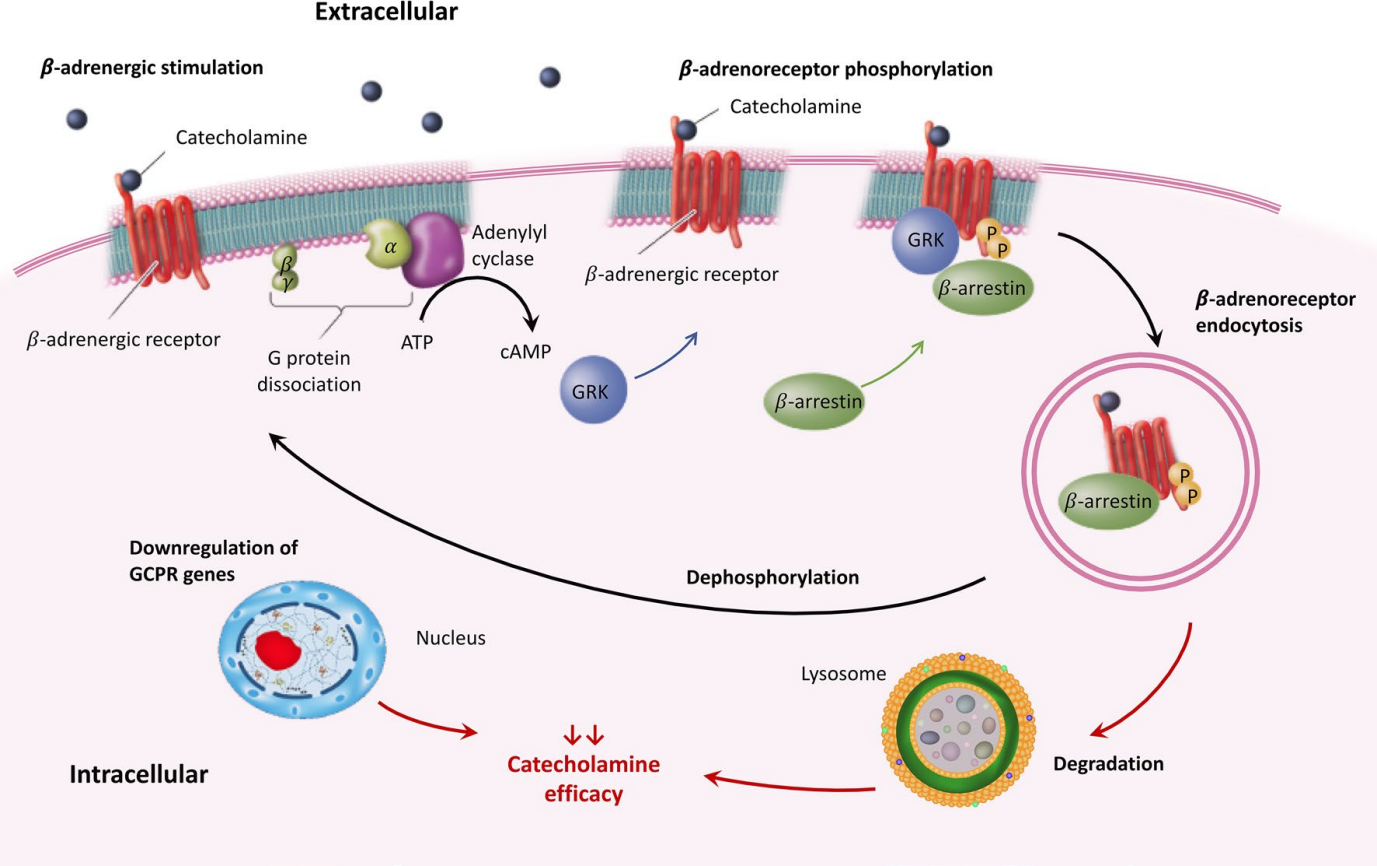
Mechanisms of adrenergic receptor activation and desensitization (here, β-adrenergic receptor). When catecholamines bind to G protein-coupled receptors (GPCR), there is a conformational change leading to dissociation of the receptors’ G protein subunits and transformation of ATP into cyclic adenosine monophosphate (cAMP). GPCR kinases (GRK) phosphorylate GPCR but only in their agonist-occupied receptor configuration. This GPCR phosphorylation by GRK enhances GPCR interaction with cytosolic proteins known as β-arrestins. These later bind to the GPCR and prevent further intracellular G-protein signaling, preventing the subsequent production of more cAMP. Increased GRK activity forces the equilibrium of β-receptors towards an inactive state. Moreover, β-arrestins promote GPCR internalization and their subsequent degradation in lysosomes. Resensitization is the process that restores the responsiveness of the desensitized receptors, through dephosphorylation by specific phosphatases and receptor recycling back to the cell membrane. Downregulation of genes encoding GPCRs also leads to reduced catecholamine efficacy, through a net loss of receptors. Of note, catecholamine binding modulates a complex cascade of enzymes and transmembrane ion channel activation, all of which are also prone to acute alteration and dysregulation due to sepsis [106–110]

Adrenergic receptors undergo frequent desensitization, a process that leads to reduced receptor responsiveness after prolonged stimulation. This action represents an important physiological “feedback” mechanism to protect the receptors against overstimulation. This desensitization process occurs by means of three mechanisms that begin soon after stimulation: phosphorylation (within seconds), endocytosis (within minutes), and downregulation (within hours) [21, 22]. Cells have elaborated a complex mechanism to dampen or turn off adrenergic stimuli through phosphorylation of their adrenergic receptors, which produces functional uncoupling of the activated receptors from their cognate G protein. Several enzymes can mediate β-adrenoreceptor phosphorylation: protein kinase A and protein kinase C phosphorylate active and inactive receptors, whereas the G protein-coupled receptor kinases (GRK) phosphorylate only agonist-occupied receptors. Phosphorylation by GRK induces adrenergic receptor binding to cytosolic proteins called β-arrestins, which further enhances the uncoupling of the receptors from their G-proteins, promoting receptor endocytosis and their subsequent degradation in lysosomes [23]. Downregulation of genes encoding G protein-coupled receptors also leads to reduced β-agonism efficacy. Finally, receptors can also undergo resensitization through specific phosphatases and be recycled back to the cell membrane (Fig. 2).

As sepsis is characterized by excessive activation of the SNS and increased levels of circulating endogenous catecholamines, the increased stimulation of adrenergic receptors activates all previously described mechanisms, leading to desensitization. These processes are enhanced further by the effect of inflammatory mediators and bacterial toxins. Indeed, several studies have shown that endotoxin, tumor necrosis factor (TNF)-α, and several interleukins, all act synergistically to reduce the responsiveness of adrenergic receptors [24].

It is important to stress that the two major pathways involved in the immune response to sepsis are the hypothalamic–pituitary–adrenal axis (HPA) and the SNS. As a consequence, most immune cells express adrenoceptors (particularly β2, but also α1-adrenergic receptors) and most functional processes in the immune system can be influenced (enhanced or attenuated) by adrenergic receptor agonists or antagonists [25] (Table 2). The complex timing of catecholamine release during sepsis has not yet been clarified and achieving the correct balance between providing cardiovascular support and causing undesirable immunomodulation remains a challenge. Indeed, the beneficial effect of norepinephrine, the most frequently used vasopressor in septic shock, is regularly questioned for several reasons: (i) it may contribute to a dysregulated immune response leading to immunoparalysis in sepsis [26]; (ii) a higher burden of endogenous [27, 28] or exogenous [29] catecholamine exposure in the ICU is associated with a worse clinical outcome; (iii) adrenergic stimulation has been extensively shown to inhibit both innate [30] and adaptative [30, 31] immune cell activity in various contexts.

**Table 2:** Mechanisms affecting the ANS that provide clinically relevant effects and may represent potential therapeutic targets during sepsis

Implicated receptors	Main endogenous and exogenous agonists	Main clinically relevant effects of receptors agonism	Potential therapeutic targets under investigation
β_1_	Norepinephrine, epinephrine, dobutamine, milrinone	Sinoatrial node and ectopic pacemaker acceleration Cardiac contractility increase Renin release by juxtaglomerular cells Potential pro-inflammatory action through macrophage activation	1) Patient “decatecholaminization” from a global standpoint: - Adrenergic burden decrease by reducing both duration and intensity (i.e., dose) exposure to exogenous catecholamines - Partial exogenous catecholamine substitution by adding/shifting to alternative non-adrenergic agents (e.g., vasopressin or levosimendan) 2) Myocardial oxygen consumption reduction and ventricular filling optimization by HR control: - Cardioselective β_1_-receptor antagonists (e.g., esmolol or landiolol) - Selective bradycardic agent (ivabradine) 3) Attenuation of β-adrenergic induced immune cell inhibition: - Cardioselective β_1_-receptor antagonists as above (e.g., esmolol or landiolol) - Less cardioselective β-blockers? - Central α2-agonist agent promotion (e.g., clonidine and dexmedetomidine) to reduce peripheral α_1_-adrenergic receptor desensitization and restore vascular tone adrenergic control
β_2_	Norepinephrine, epinephrine, dobutamine, milrinone	Arterial and venous dilation Skeletal muscle arteriole relaxation Sinoatrial node and ectopic pacemaker acceleration Cardiac contractility increase Macrophage inhibition (anti-inflammatory and immunosuppressive effects) Neutrophil activity and endothelial adhesion inhibition NK cell activity inhibition T-lymphocyte activity and proliferation inhibition (N/A for the Th2 cells) Variable influences on B-lymphocytes and antibody production	
β_3_	Norepinephrine, epinephrine, mirabegron	Variable actions on cardiac contractility Arterial and venous vasodilation Neutrophil activity and endothelial adhesion inhibition	
α_1_	Norepinephrine, epinephrine	Arterial and venous vasoconstriction Cardiac contractility slight increase Sodium reabsorption in renal tubules Glomerular arteriole (afferent > efferent) vasoconstriction Neutrophil activity and endothelial adhesion inhibition T-lymphocyte activity and proliferation inhibition	
α_2_	Norepinephrine, epinephrine	Sympatholytic effect in the CNS by presynaptic inhibition Coronary artery and arteriole vasoconstriction Sodium reabsorption in renal tubules Pro- and anti-inflammatory action on macrophages	
nACh	Acetylcholine	Macrophage activity reduction through the inflammatory reflex (spleen, liver, gut, heart)	External vagus nerve stimulation to reduce MOF and excessive pro-inflammatory reactions by the inflammatory reflex mechanism.

*β*_*1*_ beta-1 adrenergic receptors, *β*_*2*_ beta-2 adrenergic receptors, *β*_*3*_ beta-3 adrenergic receptors, *α*_*1*_ alpha-1 adrenergic receptors, *α*_*2*_ alpha-2 adrenergic receptors, *nACh* nicotinic acetylcholine receptors, *CNS* central nervous system

## The short-term ANS-mediated cardiovascular regulatory mechanism

The ANS-mediated cardiovascular regulatory system involves several afferent pathways and reflex mechanisms [19]. These mechanisms provide appropriate responses to rapid changes in cardiovascular function to maintain the BP within a physiological range of values, provide adequate blood flow to privileged organs (e.g., heart, kidneys, and brain), and redistribute it to specific regions according to metabolic demand. For these reasons, these mechanisms are known as short-term reflexes (occurring over seconds to minutes) and they include the arterial or cardiac baroreflex, the cardiopulmonary baroreflex, and the chemoreceptor reflex. Along with these short-term mechanisms, there are also long-term mechanisms of cardiovascular homeostasis (over minutes to hours or days), such as the renin-angiotensin system, to control blood volume and vascular tone.

### The arterial baroreflex

The baroreflex is the most important short-term feedback mechanism responsible for BP regulation. It is initiated by the arterial baroreceptors, usually known as high-pressure baroreceptors, which are mainly located in the aortic arch and carotid sinuses. Mechanosensitive ion channels are present on baroreceptor nerve endings, and the influx of sodium and calcium through these channels is responsible for baroreceptor depolarization during increases in BP [32]. Neural signals from arterial baroreceptors are transmitted to the nucleus of the solitary tract (NST) in the medullary area of the brainstem through two pathways: (i) signals generated by the carotid baroreceptors are transmitted through Hering’s nerve and the glossopharyngeal nerve; (ii) signals produced by the aortic baroreceptors are transmitted through the vagus nerve. The generated neural signal is transmitted to the vasomotor center through the NST in the medullary area of the brain stem. At this level, neurons project to the medullary vasomotor center, which mediates the sympathetic and parasympathetic outflows to the heart and the circulation [19]. Thus, an increase in BP induces sympathetic center inhibition, with decreased outflow to the heart and blood vessels, and parasympathetic center stimulation, with increased vagal outflow to the heart. The immediate consequence is a change in BP by vascular tone and CO modulation. The net effects are vasodilation of the veins and the arterioles and a reduction in heart rate (HR) and contractility. A decrease in arterial BP generates the opposite effects.

Importantly, baroreceptor sensitivity is not constant but depends on the BP value; specifically, baroreceptor sensitivity is maximal for BP values near the physiological range, where even a small variation in BP induces a large reflex response. Moreover, the response rate of the baroreceptors is not constant: the more rapid the BP variation, the more rapid the receptor response, regardless of the absolute BP value [33].

### The cardiopulmonary baroreflex

The cardiopulmonary baroreceptors, also known as volume receptors or low-pressure baroreceptors, are located in the cardiac atria and ventricles, in large systemic veins and in the pulmonary arteries. They behave similarly to the arterial baroreceptors as they attempt to minimize the changes in BP due to changes in blood volume, mostly contained in the veins. Indeed, changes in atrial pressure are often associated with changes in venous return, which is altered by changes in CO and blood volume. The atria contain receptors activated by the tension developed during atrial contraction and receptors activated by the stretch of the atria during atrial filling; when stimulated, they send impulses up the vagal fibers to the vagal center in the medulla. Consequently, sympathetic activity is decreased to the kidney and increased to the sinoatrial (SA) node. These changes in sympathetic activity result in increased renal blood flow, diuresis, and HR.

Cardiopulmonary receptors stimulation can also lower BP by inhibiting the vasoconstrictor center in the medulla and inhibits angiotensin, aldosterone, and vasopressin (ADH) release; interruption of the reflex pathway has the opposite effects. Changes in diuresis elicited by changes in cardiopulmonary baroreceptor activation are important in the regulation of blood volume. For example, hypovolemia enhances sympathetic vasoconstriction in the kidney and increases secretion of renin, angiotensin, aldosterone, and ADH [19]. However, compared to the changes induced by the arterial baroreflex, cardiopulmonary receptor-induced variations in HR have a much smaller effect [34].

It is important to note that the direct effect of the cardiopulmonary reflex on contractility has not yet been clarified. The Bainbridge reflex is, indeed, still a matter of debate. This reflex, in contrast with the two types of baroreflex discussed earlier, consists of a transient increase in HR in case of a rapid increase of blood volume, leading to transient tachycardia concomitant with high right atrial pressures. The Bainbridge reflex has been demonstrated in animals like dogs and rats, but is not fully understood in humans; nonetheless, some studies suggest a possible role for this reflex in cardiovascular regulation when there are large variations in venous return [35, 36].

### The chemoreflex

The chemoreceptors represent another mechanism involved in the regulation of BP and respiratory activity. These receptors are located peripherally (carotid bodies and aortic arch) and centrally (respiratory center of brain medulla) and they contribute to maintaining the arterial pH and both arterial partial pressures of oxygen and carbon dioxide within appropriate physiological ranges. Different from baroreceptors, chemoreceptors respond to chemical stimuli. In particular, hypercapnia detected by central chemoreceptors or hypoxia detected by peripheral chemoreceptors leads to an increase in respiratory rate and tidal volume, mediated by the neural respiratory center. Anatomically, the chemoreceptors transmit afferent signals through the vagus nerve to the NST where the respiratory center is located. Furthermore, the respiratory center also stimulates the vasomotor center; hence the net effect is a concomitant increase in arterial BP by means of vasoconstriction, although this reflex has a limited effect compared to the arterial baroreflex [19, 37]. The chemoreceptor reflex has been widely studied and reviewed [38, 39] and can be summarized as an inhibitory feedback process that interacts closely with the baroreflex [40, 41].

## ANS-mediated cardiovascular regulation in septic shock

### Sympathetic overstimulation

In septic shock, cardiovascular regulatory mechanisms responsible for counteracting the hypotensive stress induced by the septic immune response are impaired. The normal compensatory response to hypotension includes increased sympathetic outflow to the heart and peripheral vessels to restore BP to normal values [42]. Several studies have demonstrated dysfunction of the sympathetic branch of the ANS in septic shock, i.e., a maladaptive response to the hypotensive and inflammatory stress, which leads to impaired autonomic control of the heart and vessels, contributing to circulatory failure. The exact pathophysiological mechanism underlying this autonomic imbalance is not yet clear, but excessive, uncontrolled or prolonged SNS activation and/or inappropriate downregulation of the PSNS are key factors [42, 43]. Overall, these alterations are generally referred to as autonomic dysfunction or dysautonomia.

Life-threatening illness, such as septic shock, is one of the most potent stimuli of the SNS and extensive sympathetic activation is documented by elevated concentrations of endogenous circulating catecholamines—i.e., plasma epinephrine and norepinephrine—during shock and persisting into the post-resuscitation phase. Persistently high plasma catecholamine concentrations have been demonstrated to be associated with increased morbidity and mortality in critically ill populations [27, 29] and, in particular, in patients with septic shock [28, 44, 45], compared to progressive normalization of concentrations. Protracted and overwhelming adrenergic stress may exceed in time and scope the beneficial short-term compensatory effects. The entity of damage depends on the vulnerability of the different organs to adrenergic overstimulation and to the presence of coexisting chronic diseases. For example, the heart, which has abundant β-adrenergic receptors, seems to be most susceptible to sympathetic overstimulation, with detrimental consequences such as impaired diastolic function, tachyarrhythmia, myocardial ischemia, vasospasm, impaired coronary microcirculation, stunning, and apoptosis [20]. Other organs are also affected by adrenergic stress, with associated consequences such as pulmonary edema (with subsequent right ventricular dysfunction/failure), increased thrombosis formation, gastrointestinal hypoperfusion, immunosuppression, increased cell energy expenditure and microvascular dysfunction [42, 43, 46].

### Dysfunctions of the afferent, central and efferent ANS pathways

In addition to its overstimulation, the SNS is widely disturbed by inflammatory mediators during sepsis and septic shock, with dysfunction of afferent, central, and efferent pathways, including massive desensitization of its adrenergic receptors.

Pro-inflammatory cytokine release and overproduction of nitric oxide (NO) have been shown to down-regulate adrenergic receptors [47–49], including the cardiac β1-adrenergic receptors, which could contribute to the reduced cardiovascular responsiveness to adrenergic stimuli that is frequently observed in septic shock [50]. Downregulation of α1-adrenergic receptor gene expression during sepsis has been demonstrated in vivo and in vitro as a result of pro-inflammatory cytokine release and was associated with circulatory failure [51].

ANS chemoreceptor dysregulation also occurs in sepsis. In vitro and animal experiments in endotoxic shock have reported that inflammatory mediators activate the chemosensitive glomus cells of the carotid body, leading to increased and excessive respiratory activity [52], explaining in part why a high respiratory rate is a hallmark of sepsis onset. In a small clinical study in patients with multiorgan failure (MOF), as a result of sepsis in two thirds, cardiac chemoreflex sensitivity was blunted in proportion to disease severity (i.e. the higher the severity score, the more the variations in arterial oxygen partial pressure were not followed by the expected variations in heart rate, as in the controls subjects) [53].

Continuous communication among all vital organs is guaranteed through neural signals mediated by the ANS, which allows constant adaptation to the different physiological and pathological conditions. In the fundamental work by Godin and Buchman [54], this communication was considered in terms of “coupled oscillators”: i.e., the vital organs could be seen as biological oscillators that are coupled to one another. The overwhelming inflammatory reaction associated with sepsis disrupts this communication by initiating uncoupling of these oscillators. Progression into MOF may reflect further progressive uncoupling that becomes irreversible, whereas recovery may be related to restoration of oscillator coupling. Bacterial toxins and inflammatory sepsis mediators can alter neural reflexes and consequently cause uncoupling of these inter-organ connections [55]. However, the exact mechanism responsible for this autonomic dysfunction has not been determined, and it is not clear whether it is due to a reduction in central vasomotor activity, to altered peripheral neuroeffector transmission, or depressed end-organ responsiveness as a result of desensitization.

Several studies documented how baroreflex often plays the major driver role of this autonomic imbalance. Indeed, in animal models of septic shock, reduced baroreflex function has been associated with reduced survival times [9, 56, 57]. This sepsis-related dysfunction of BP regulation may involve the baroreflex arch at several levels, including (i) a reduction in baroreceptor sensitivity; (ii) a shift in the baroreflex set point to lower levels of BP with impaired efferent sympathetic activity; (iii) reduced responsiveness of the target organ.

A recent study on endotoxic shock induced in rats by *Escherichia coli* lipopolysaccharide (LPS) infusion, demonstrated that the impairment of the baroreflex occurred almost immediately after induction of the inflammatory reaction, without changes in BP, and persisted after the LPS infusion had been stopped [5]. The authors hypothesized a direct effect of LPS through the production of NO and reactive oxygen species (ROS) (extrapolating their hypothesis from similar observations in a carotid arteriosclerosis animal model [58]). Circulating cytokines released after LPS infusion may also play an important role since they were shown to induce inflammation of the carotid body with a consequent reduction in arterial distensibility and, therefore, of baroreceptor engagement [59].

The limited knowledge about the role of the SNS on cardiovascular system control during sepsis is partly related to the difficulty in directly assessing sympathetic activity. Indirect measures of autonomic activity, such as HRV, do not enable discrimination between compromised sympathetic outflow at the central level or at the level of peripheral neuroeffector transmission. Nevertheless, sympathetic outflow can be directly measured in animal experiments through microneurographic measurements of muscle, cardiac, or renal sympathetic nerve activity (MSNA, CSNA, and RSNA, respectively). These measures enable quantification of the centrally regulated sympathetic outflow to different peripheral organs and thus for the ANS-mediated mechanisms activated in response to sepsis and septic shock to be unraveled [60, 61]. Ramchandra et al. [61] studied the changes in regional sympathetic activity following *E. coli* infusion in conscious sheep. They reported an increase in CSNA highly correlated with the increase in HR, and a transient decrease in RSNA during the first 3 h after infusion followed by a sustained increase. Vayssettes et al. [62] also reported a strong correlation between increased RSNA and tachycardia in anesthetized rats after LPS infusion; by contrast, arterial baroreceptor denervation had a minimal effect on the RSNA increase induced by LPS. However, in healthy human volunteers, Sayk et al. [60] showed that injection of endotoxin was associated with suppressed MSNA, concomitant with an increased HR and a blunted MSNA baroreflex-mediated response. The increased HR did not change with BP modulation, indicating that HR was uncoupled from baroreflex regulation. This finding suggests that the immune response in sepsis directly suppresses sympathetic outflow to the muscle vascular bed via central nervous mechanisms, leading to blunted baroreflex sensitivity [60].

Together these studies highlight that altered baroreflex control drives the sympatho-excitation elicited by sepsis, supporting previous research that demonstrated that the sympathetic activation observed in septic shock was greater than that expected with the simple unloading of baroreceptors because of the hypotensive stress [63, 64].

### Inflammatory reflex

**Fig. 3:**
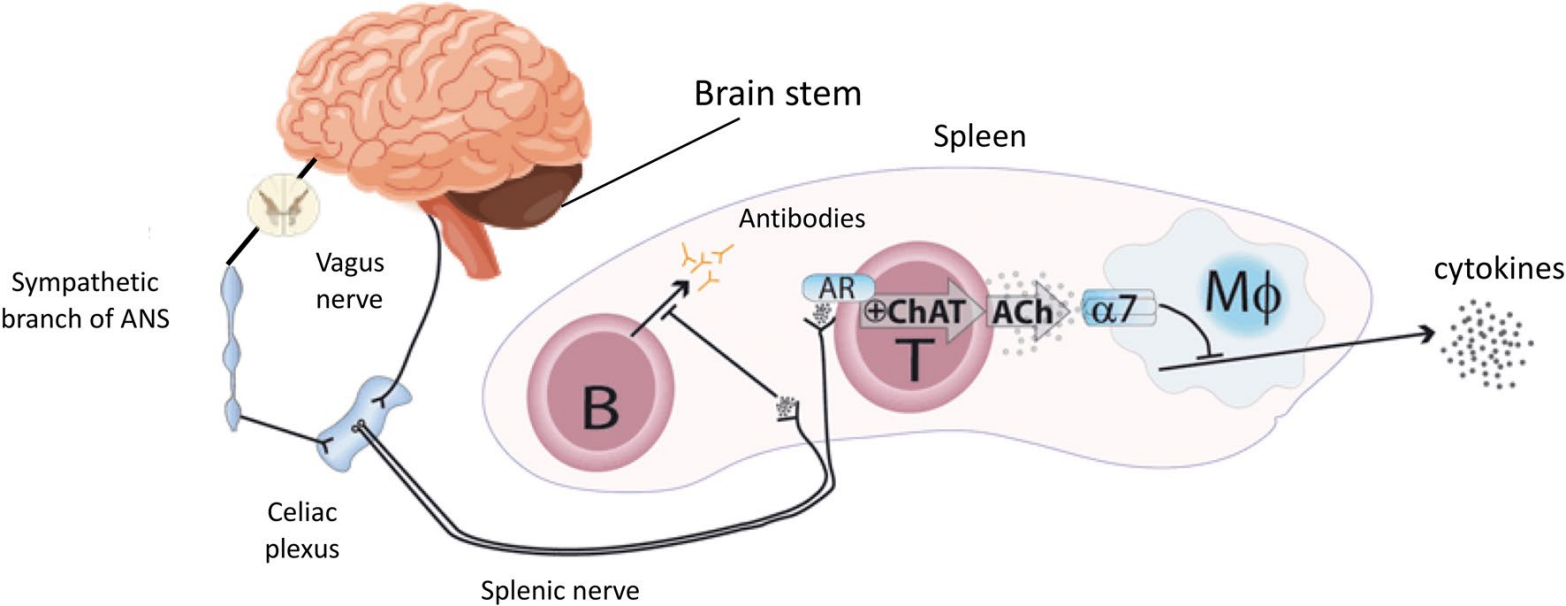
Illustration of the inflammatory reflex (here, in the spleen). Efferent signals from the brain stem travel through the efferent vagus nerve to the celiac plexus, which also receives input from the sympathetic branch. The catecholaminergic splenic nerve arises in this celiac plexus and projects inside the spleen, where its terminal fibers reach T and B lymphocytes. Efferent signals in the vagus nerve activate the splenic nerve, which releases norepinephrine in the spleen, activating the choline acetyltransferase‐expressing T-lymphocytes (ChAT) through their adrenergic receptors (AR). ChAT then release acetylcholine (ACh), which acts on α7 subunits (α7) of the nicotinic acetylcholine receptor on macrophages (*M*_Φ_), suppressing further release of tumor necrosis factors (TNF). Activation of the splenic nerve also stops B‐lymphocyte migration and inhibits their antibody production. Adapted from [111]

The term “inflammatory reflex”—or sometimes “cholinergic anti-inflammatory response”—highlights the increasing awareness that the PNS reflexly regulates the inflammatory response in real-time by inhibiting tissue macrophage activation [65, 66], just as it controls HR and other vital functions. Briefly, efferent signals in the vagus nerve lead to acetylcholine release in target organs of the reticuloendothelial system, such as the liver, heart, spleen, and gastrointestinal tract. Inside these organs, acetylcholine interacts with its nicotinic receptors on tissue macrophages to inhibit further release of TNF, interleukin (IL)-1, high mobility group B1 (HMGB1) and other pro-inflammatory cytokines. Of note, this cholinergic stimulation of macrophages does not reduce anti-inflammatory cytokine release (e.g., IL-10) [65]. Figure 3 illustrates this mechanism for the spleen.

Important work by Fairchild and colleagues [67] has confirmed that the PNS and immune system interaction is actually bilateral, with vagal efferent signaling but also sensory afferent signaling, supporting the idea of a complete reflex loop. Indeed, mice exposed to pathogens by induction of peritonitis, demonstrated activation of both vagal efferent and afferent signaling pathways; these latter pathways activated cholinergic visceromotor neurons including the dorsal motor nuclei of the solitary tract in the brain stem.

The crucial role of PNS-mediated inflammatory modulation in sepsis has been illustrated by several studies with research demonstrating that electrical stimulation of the efferent vagus nerve inhibits the pro-inflammatory cytokine cascade, limiting the potentially damaging systemic inflammatory response and improving outcomes in animal models of endotoxic shock (opposite results being observed after vagotomy) [66, 68, 69]. This beneficial effect of the inflammatory reflex extends beyond infectious disease and pathogen-induced inflammation, as it has been shown, for example, to produce cardioprotection after acute myocardial infarction, significantly reducing myocardial ischemia/reperfusion injuries in pre-clinical investigations [70, 71].

## Indices of autonomic dysfunction

**Fig. 4:**
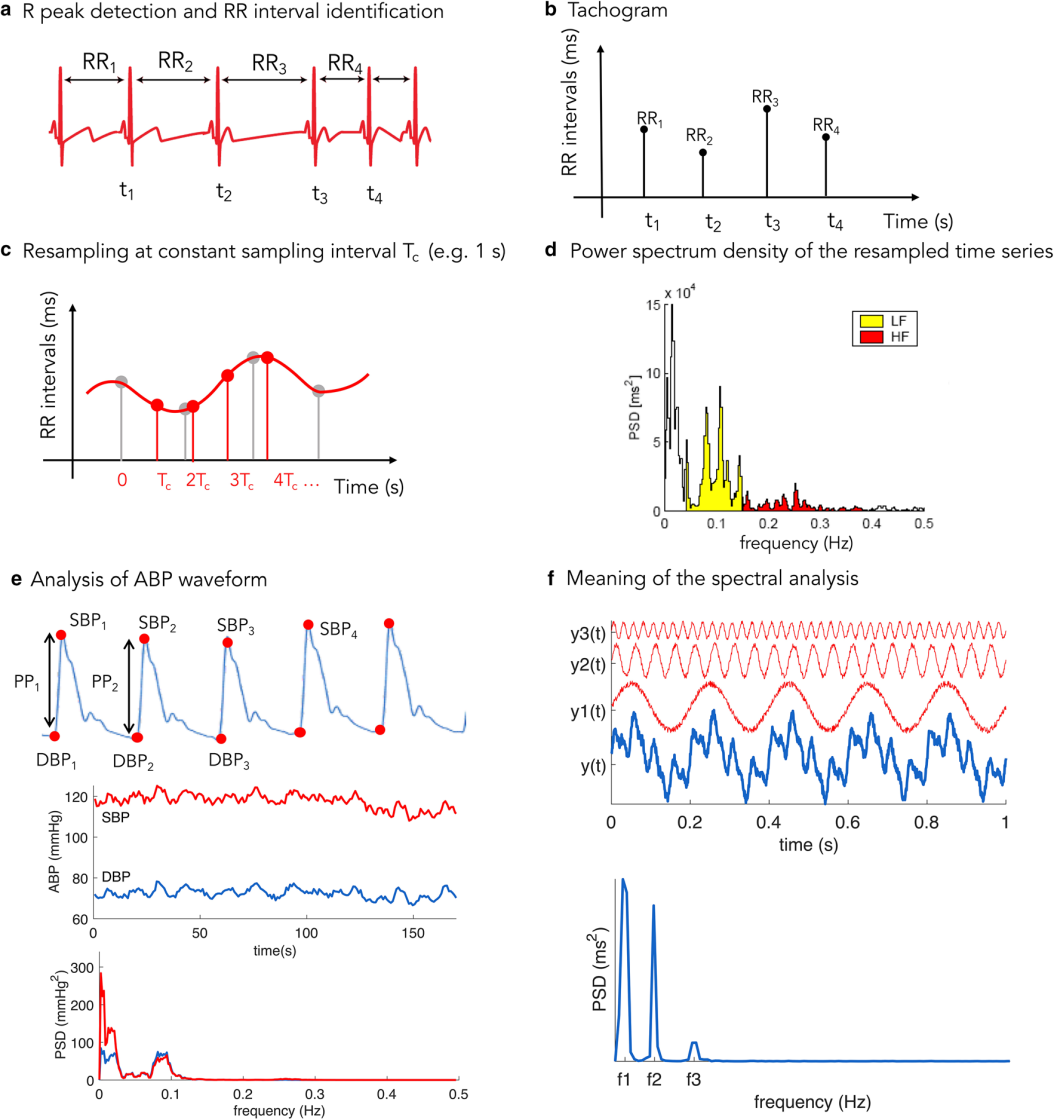
Basic concepts to estimate heart rate and blood pressure variabilities. **a** Identification of R peaks in the ECG signal—in sinus rhythm—permits assessment of the RR interval duration for consecutive beats. **b** The tachogram displays RR intervals for each beat; this time series analysis permits a first visual inspection of the variability in RR interval duration, computed as the mean RR interval and its standard deviation. **c** Before the spectral analysis, the time series needs to be resampled at evenly spaced time intervals (*T*_c_ = sampling interval which corresponds to *f*_c_ = 1/*T*_c_ sampling frequency). **d** Power spectrum density (PSD) computation and spectral analysis: as the parasympathetic nervous system (PNS) acts at higher frequencies than the sympathetic nervous system (SNS), the region associated with high frequency (HF, 0.15–0.4 Hz) estimates the PNS contribution to RR interval modulation, whereas the low frequency (LF, 0.04–0.15 Hz) represents mainly the SNS contribution; LF and HF indexes represent the PSD areas in those bands. **e** Similar to HRV, beat-to-beat series of systolic (SBP) and diastolic (DBP) blood pressure values lead first to the time domain analysis and, after resampling, to PSD computation and spectral analysis (NB: in case of blood pressure, HF variations are not related to the PNS, but mainly to the respiratory activity). The simplest way to assess the baroreflex sensitivity consists of calculating the ratio between LF components of both SBP and RR series, in their respective PSD computation. **f** The spectral analysis permits to disentangle the individual contributions of several modulators (in this example, the signal *y*(*t*) is composed of three harmonics and the PSD can clearly identify those harmonics with the low-frequency signal, *y*1(*t*), contributing more than the other two)

Clinical tools that can be used to characterize autonomic dysfunction include HRV, BPV, baroreflex sensitivity (Fig. 4) and, less frequently, chemoreflex sensitivity [72–76]. A reduction in physiologic variability, measured with the standard indices of HRV, BPV and the BRS index, has been demonstrated to be directly correlated with septic shock severity and mortality in several experimental and clinical studies [7, 8, 77–79].

Reduced HRV and compromised vagal activity at the cardiac level have been documented in patients with septic shock. The association between inflammation and HRV has been investigated in a rodent endotoxemia model. The authors reported that LPS-induced cytokine elevation was closely linked to HRV changes, as the major cytokine peaks were concomitant with maximal HRV depression [80]. In another study, the high levels of circulating catecholamines reported in septic shock were inversely correlated with indices of HRV [78]. Explanations for this finding may be that, similar to the situation in patients with heart failure, high sympathetic drive may lead to saturation of low-frequency oscillatory systems [81], or that excessive concentrations of circulating catecholamines may compromise central autonomic control [10, 60].

In a recent study by our group [82], analysis of cardiovascular variables in an experimental polymicrobial septic shock model in pigs suggested a pattern of autonomic dysfunction typical of septic shock. There was a significant positive correlation between an increase in aortic arterial stiffness and depressed vagal activity, together with decreased total peripheral resistance (TPR), decreased Windkessel time constant, τ, and impaired sympathetic activity at the peripheral sites. Interestingly, this abnormal condition generated by septic shock did not resolve after resuscitation (i.e., volume expansion and norepinephrine perfusion) and correction of hypotension. Although global hemodynamic markers were restored by the resuscitation maneuvers, the indices of cardiovascular function mediated by the ANS were still impaired, suggesting that the baseline homeostatic control had not completely recovered, similar to findings in an earlier study in an experimental model of hemorrhagic shock [83].

In another study by our group [84], the early response to standard resuscitation in septic shock patients was analyzed; patients were stratified according to the change in the sequential organ failure assessment (SOFA) score within 48 h of ICU admission. All the patients reached resuscitation goals, i.e., a mean arterial pressure (MAP) > 65 mmHg, but only the patients who significantly improved their SOFA score (responders) showed an increase in the BP oscillations associated with the sympathetic outflow. In the non-responders, the fluid balance was higher and, despite a higher dose of vasopressors, there was no increase in the BP oscillations associated with the sympathetic outflow. This observation may indicate poor responsiveness to the vasopressor therapy in these patients, despite similar BP values to the SOFA score responders.

From these results, BP variability, HRV, and baroreflex analyses can be considered valuable tools to understand the responsiveness of patients to sympathomimetic drugs and/or fluid administration and to identify patients with a worse prognosis who require more invasive or frequent monitoring. Resuscitation strategies should consider the balance of sympathetic and autonomic tone, considering the potential role of redirecting and maximizing sympathetic activity [43].

## Therapeutic interventions to modulate the ANS in septic shock

Table 2 summarizes some innovative therapeutic paradigms for modulating ANS in septic shock, although more robust clinical trials are needed to confirm whether they have beneficial effects on clinically-relevant outcomes.

### Cardioselective β1-blockers and ivabradine

Although the concept of “adrenergic toxicity” in acute illness and in septic shock is well established in the scientific literature, there are no clear clinical recommendations for ANS modulation. For example, tachycardia persisting despite fluid and vasopressor administration is a hallmark of septic shock and an independent risk factor for increased mortality [85, 86]. The consequences of excessive tachycardia are multifactorial and include: (i) less diastolic time available for ventricular filling (i.e., impaired diastolic function) and for left ventricular myocardial coronary perfusion, while myocardial oxygen consumption is actually increased (i.e., potential functional ischemia); (ii) a higher risk of developing tachyarrhythmia; (iii) a certain degree of heart failure by cellular exhaustion when tachycardia is excessively high and prolonged (i.e., tachycardiomyopathy). One of the sources of this persistent tachycardia has been demonstrated to be protracted adrenergic stress at the cardiac level, which exceeds in time and degree the beneficial short-term compensatory effects [87].

For this reason, drugs that attenuate tachycardia are currently under investigation, assuming that the reduction in HR will be hemodynamically compensated for by longer ventricular filling, with higher stroke volume, and hence, a reasonably preserved CO. To date, the most advanced investigations involve ultra-short acting cardioselective β1-blockers, such as esmolol or landiolol. Esmolol administration has been tested in animal experiments [88, 89] and in a single center, open-label randomized clinical trial in septic shock patients with persistent tachycardia despite 24 h of full resuscitation [90]. Esmolol was associated with reduced HR and an unexpected faster vasopressor weaning together with an improvement in the 28-day mortality. This result still needs confirmation in a larger, multicenter clinical trial, however, because a recent attempt to replicate these observations was disappointing, mostly from a safety standpoint because of insufficient stroke volume compensation [91]. This risk related to the negative inotropic effect of esmolol was previously illustrated in another septic shock animal experiment, in which esmolol also had a global hypotensive effect [92]. Landiolol has also been studied in septic shock patients. In a multicenter Japanese randomized clinical trial with a similar design, landiolol was associated with HR reduction but with no statistically significant reduction in mortality, although mortality was not the primary endpoint of the study [93].

To overcome the negative inotropic effect of beta-blockers, another negative chronotropic drug was recently proposed, ivabradine, which specifically inhibits the pacemaker current (funny current, If) of the sinoatrial node cells, resulting in therapeutic HR lowering, without any negative inotropic or hypotensive side effects [94]. In acutely ill patients, ivabradine has been shown to effectively lower HR, but a beneficial effect on more relevant outcomes, such as major adverse cardiovascular events and mortality, has yet to be demonstrated [95]. A large randomized clinical trial aimed at controlling HR with ivabradine in septic shock is currently ongoing (IRISS trial; NCT04031573).

In addition to the already mentioned pathophysiological mechanisms (e.g., an attenuation of impairments in ventricular filling and myocardial perfusion), a possible mechanism explaining some of the beneficial effects of these β1 selective antagonists is an indirect immunomodulatory effect. These agents were shown to stimulate vagal nerve activity in an endotoxemia animal model [96] and to preserve normal splenic T-lymphocyte counts in an animal sepsis model [97]. Moreover, in an animal endotoxemia model, landiolol infusion suppressed the high-mobility group box 1 (HMGB-1) expression, while improving lung injury and cardiac function [98].

### Central α2-agonists

In addition to peripheral adrenergic modulation, it is important to mention the more recent interest in pharmacologic interventions acting on central α2-adrenergic receptors located in the brain and, in particular, on vascular pre-junctional terminals, where they can inhibit the release of norepinephrine in a form of negative feedback. Although conventional understanding of α2-agonists has considered these drugs as antihypertensive agents (e.g., clonidine), robust evidence now highlights their role in CNS outflow as light sedative agents with a positive impact on peripheral adrenergic receptor regulation (e.g., dexmedetomidine). Intravenous infusion of central α2-agonists in animal models of septic shock almost completely restored vascular responsiveness to catecholamines and angiotensin to pre-septic conditions [99]. This raises the hypothesis that central α2-agonists inhibit central sympathetic outflow and consequently reduce the high levels of norepinephrine released at sympathetic nerve terminals. This effect would reduce α1-receptor desensitization and transform it into a gradual resensitization. In other words, vascular α1-receptors that were down-regulated in septic shock become progressively up-regulated on the administration of α2-agonists [100]. This hypothesis also suggests that the more the α1-receptors are desensitized by high concentrations of endogenous catecholamines (e.g., in refractory septic shock), the larger the expected improved responsiveness to vasopressors. In recent studies and subgroup analyses, dexmedetomidine appeared to be associated with lower vasopressor requirements to maintain the same MAP target in septic shock [101, 102].

### Vagal stimulation

In the last few years, a new field, “bioelectronic medicine”, has been rapidly evolving, with the discovery and development of innovative nerve stimulating and sensing technologies to diagnose and regulate biological processes and treat disease. The idea is that by manipulating the neural signals, it may be possible to change the way physicians treat pathological conditions, including autoimmune disorders (e.g., rheumatoid arthritis), inflammatory pathologies (e.g., Crohn’s disease), and potentially also sepsis and bleeding [65].

This new technology has been applied to the field of the “inflammatory reflex”—described earlier—and exploited in numerous studies, which have shown the beneficial effect of vagal stimulation in treating animal models of endotoxemia and septic shocks [68, 103, 104]. In a recent experimental swine study of polymicrobial sepsis [105], animals were divided into three groups: sham, sepsis, and sepsis with vagus nerve cervical electrical stimulation (initiated 6 h after peritonitis induction). Animals treated with vagal nerve stimulation required less fluid and norepinephrine administration to achieve the resuscitation targets. Vagus nerve stimulation reduced the number of activated macrophages and partially or completely prevented the development of hyperlactatemia, hyperdynamic hemodynamic status, septic cardiomyopathy, and cardiac mitochondrial dysfunction.

## Conclusion and future perspectives

ANS dysfunction has an impact on the responsiveness of septic shock patients to fluids and to adrenergic drug administrations. BPV, HRV, and baroreflex sensitivity can be considered valuable tools to understand and quantify the ANS dysregulation associated with the potential loss of cardiovascular homeostasis. They could therefore be integrated into standard hemodynamic monitoring systems, to strengthen our ability to anticipate deterioration in patient condition, or to better modulate some therapies, such as vasopressors, including their dose and selection (e.g., whether or not to give adrenergic agents). Adrenergic vasoactive drug prescriptions should be tailored and personalized, to obtain a better balance between cardiovascular support and undesirable immunomodulation during septic shock.

A better understanding of ANS-mediated control of the cardiovascular system during septic shock may translate into future therapeutic expansion towards negative chronotropic agents (e.g., beta-blockers or ivabradine) or central α2 agonists. Availability of novel technologies, such as noninvasive transcutaneous vagus nerve stimulation, in the clinical setting, will help pave the way for other innovative therapies to selectively stimulate the nervous system.

## Data Availability

Not applicable.

## Abbreviations

ANS: Autonomic nervous system
BP: Blood pressure
BPV: Blood pressure variability
CO: Cardiac output
CSNA: Cardiac sympathetic nerve activity
GRKs: G protein-coupled receptor kinase
HR: Heart rate
HRV: Heart rate variability
ICU: Intensive care unit
iNOS: Inducible nitric oxide synthase
LPS: Lipopolysaccharide
MAP: Mean arterial pressure
MOF: Multi-organ failure
MSNA: Muscle sympathetic nerve activity
NO: Nitric oxide
NST: Nucleus of the solitary tract
PNS: Parasympathetic nervous system
PP: Pulse pressure
ROS: Reactive oxygen species
RSNA: Renal sympathetic nerve activity
SAD: Sino aortic denervation
SNS: Sympathetic nervous system
SOFA: Sequential organ failure assessment
SV: Stroke volume
TPR: Total peripheral resistance
